# Application of 3D-Printed Personalized Guide in Arthroscopic Ankle Arthrodesis

**DOI:** 10.1155/2018/3531293

**Published:** 2018-09-12

**Authors:** Xiaojun Duan, Peng He, Huaquan Fan, Chengchang Zhang, Fuyou Wang, Liu Yang

**Affiliations:** ^1^Center for Joint Surgery, Southwest Hospital, Third Military Medical University (Army Medical University), Chongqing 400038, China; ^2^Chongqing Institute of Optics and Machines, Chongqing 401122, China

## Abstract

**Objective:**

To accurately drill the Kirschner wire with the help of the 3D-printed personalized guide and to evaluate the feasibility of the 3D technology as well as the outcome of the surgery.

**Methods:**

Patients' DICM data of ankle via CT examinations were introduced into the MIMICS software to design the personalized guides. Two 2mm Kirschner wires were drilled with the help of the guides; the C-arm fluoroscopy was used to confirm the position of the wires before applying the cannulated screws. The patients who underwent ankle arthrodesis were divided into two groups. The experimental group adopted the 3D-printed personalized guides, while the control group received traditional method, i.e., drilling the Kirschner wires according to the surgeon's previous experience. The times of completing drilling the Kirschner wires to correct position were compared between the two groups. Regular follow-ups were conducted to statistically analyze the differences in the ankle fusion time and AOFAS scores between the two groups.

**Results:**

3D-printed personalized guides were successfully prepared. A total of 29 patients were enrolled, 15 in the experimental group and 14 in the control group. It took 2.2 ± 0.8 minutes to drill the Kirschner wires to correct position in the experimental group and 4.5 ± 1.6 minutes in the control group (*p*=0.001). No obvious complications occurred in the two groups during and after surgery. Postoperative radiographs confirmed bony fusion in all cases. There were no significant differences in the fusion time (*p*=0.82) and AOFAS scores at 1 year postoperatively between the two groups (*p*=0.55).

**Conclusions:**

The application of 3D-printed personalized guide in assisting the accurate drilling of Kirschner wire in ankle arthrodesis can shorten the operation time and reduce the intraoperative radiation. This technique does not affect the surgical outcome.

**Trial Registration Number:**

This study is registered on www.clinicaltrials.gov with NCT03626935.

## 1. Introduction

Ankle arthrodesis is a reliable surgical method for end-stage ankle arthritis [1–3]. Arthroscopic ankle arthrodesis, with advantages of smaller incisions, higher fusion rates, and fewer complications, has gradually gained popularity in recent years. This technique mainly uses cannulated screws for fixation [4–8]. In addition to proper procedure of the articular surface, the position of the screw is also of great importance. The satisfactory position of the screw is based on the accurate drilling of Kirschner wire. Traditionally, this process is done with the previous experience of the surgeons. C-arm fluoroscopy is constantly used during the surgery to confirm the position of Kirschner wire, which not only wastes the operation time but also increases the adverse effects of intraoperative radiation.

A new strategy to solve the above problem is to apply personalized guides that are highly consistent with the patients' anatomical data. As a new rapid prototyping technology, 3D printing technology and its medical application are gaining popularity [9–14]. The authors utilized the patients' CT data to obtain their ankle morphology and structure to prepare the 3D-printed personalized guides. With the help of the guides, the cannulated screws were successfully implanted according to the preoperative plan. In this retrospective study, we tried to investigate the efficacy of 3D-printed personalized guide implemented in the arthroscopic ankle arthrodesis to provide further reference for its future promotion.

## 2. Materials and Methods

### 2.1. Participants

The inclusion criteria were (1) primarily diagnosed as traumatic, degenerative, and rheumatoid arthritis; (2) weight-bearing radiographs showing that the tibiotalar surface angle less than 15°; (3) course of disease more than 6 months and pain still existing after conservative treatment. The exclusion criteria were (1) significant degeneration in the subtalar and talonavicular joint; (2) comorbidity with ischemic necrosis of the talus; (3) ankle stiffness and lack of mobility that made it impossible to place the arthroscope; (4) ankle tuberculosis, infection, tumor, and Charcot joint disease; (5) obvious coagulation disorder.

Twenty-nine patients between Jan 1, 2014, and Jun 30, 2016, were enrolled, 15 males and 14 females, aged 55 ± 12 years (range: 41-70 years). There were 12 cases of left ankle and 17 cases of right; all received unilateral surgery. The mean duration of ankle pain was 4.3 years (range: 1-25 years). All surgeries were performed by the same senior surgeon in the center (X. Duan). Those whose radiographs of anteroposterior view showed varus or valgus tibiotalar surface angle of more than 15° were recommended to receive open surgery and were not included.

### 2.2. Preoperative Imageological Examination

Routine radiographs of anteroposterior and lateral view in standing position were obtained preoperatively. Measurement of the tibiotalar surface angle was conducted to determine whether the ankle joint space was narrow. Artoscan C (0.2T, Esaote, Italy) was used to scan the ankle joint and help determine the cartilage and ligament injury, as well as the arthritis in subtalar and talonavicular joint.

### 2.3. Preoperative Preparation of 3D-Printed Personalized Guide

**Figure 1:**
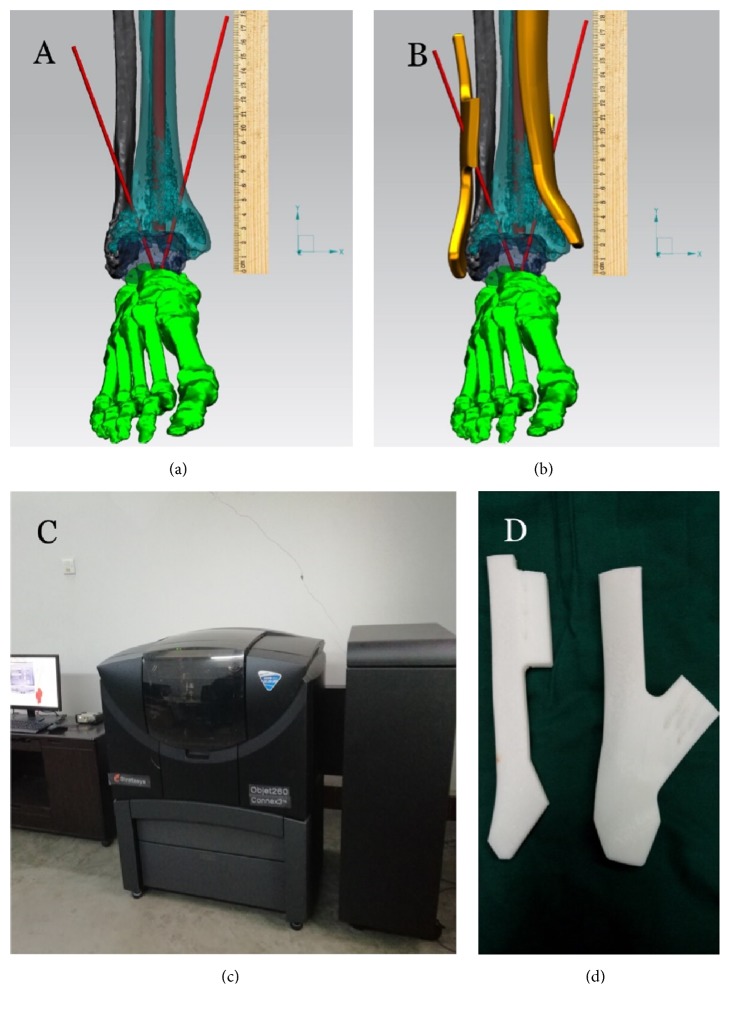
**Design and preparation of the 3D-printed personalized guide. (a, b)** Computer software-assisted design of personalized ankle arthrodesis guide (the red lines in (a) demonstrate the positioning of the Kirschner wires that drill through, and the yellow parts in (b) are the guides).** (c)** Guide data converted into STL format and imported into the 3D printer for preparation.** (d)** The 3D-printed personalized guides.

Three-dimensional thin-layer CT scan (Siemens AG, Germany) of the ankle was conducted with the CT scan slice thickness of 1mm. DICM data was extracted and imported to the MIMICS software to reconstruct the 3D data of the ankle and surrounding tissues. The reconstructed data was imported into SIEMENS NX 3D software to design the medial and lateral guides for ankle arthrodesis. The distal end of the medial guide should be placed at the surface of the medial malleolus, with the posterior edge of the guide close to the posterior edge of the tibia. The longitudinal axis of the guide should be parallel to the longitudinal axis of the tibia. The guide had guide hole of about 2mm in diameter, from which the 2mm Kirschner wire could be drilled from the posterior edge of the tibia. The wire should form a 65° angle with the tibiotalar joint after reduction in the coronal plane, as well as a 30° angle with the tibial axis in the sagittal plane [3]. More guide holes were made near the best angle for intraoperative use in case the position of Kirschner wire needed to be adjusted. For the lateral guide, its distal end should be placed at the tip of the lateral malleolus with close contact with the distal end of the fibula. The guide hole was designed at the anterior edge of the guide so that the Kirschner wire drilled through could be parallel to the fibular axis in the sagittal plane, forming a 65° angle with the tibiotalar joint in the coronal plane. The data of the designed guide was converted into STL format and imported into the 3D printer (Stratasys Objet260 Connex3™, USA). Polylactic acid (Tiertime, China) was used as the raw material to print the medial and lateral guides (Figure 1). The guides were sterilized with ethylene oxide and sealed for the surgery.

### 2.4. Surgical Techniques

The preferred anesthesia method was nerve block anesthesia; epidural anesthesia or general anesthesia was also acceptable. Patients were in conventional supine position with thigh tourniquet pressured 280-300 mmHg. Leg holder was used on the affected side. The ipsilateral hip was moderately elevated, with flexion of the affected knee and hip joint; the ankle was kept in neutral position. After routine sterilization and draping, soft ankle traction device was applied. The healthy limb was placed flat on the operating table. C-arm fluoroscopy was connected to ensure convenient utilization during the surgery.

Ankle paracentesis was conducted and 20mL of saline was infused. Anteromedial incision of the ankle was established for exploration, and then the anterolateral portal was established under arthroscope. Lesions of the ankle cavity were explored, and the residual articular cartilage was debrided with 4.5mm soft tissue blade and curette. Sclerosed bone was slightly removed using a bony electric grinder to make it “fresher”. Microfracture was conducted in part of the sclerotic bone to facilitate the fusion [3]. Osteophyte in the anterior part of the tibia was removed, which was conducive to arthroscopic exploration and ankle reduction, but the osteophyte did not need to be completely removed.

With the gradual debridement of the articular cartilage, the joint space became more obvious, and the operating space in the joint cavity became larger for the arthroscopy. Mild varus or valgus deformity of the ankle could be moderately adjusted during ankle arthrodesis. After the articular surface treatment was completed, the guides were placed close to the skin. With the help of the medial guide, a 2mm Kirschner wire was drilled from the posterior edge of the tibia; the drilling should be stopped when the tip of the Kirschner wire was seen just entering the ankle joint under arthroscope. With the help of the lateral guide, another 2mm Kirschner wire was drilled according to preoperative plan. Then the arthroscope was pulled out and the ankle reduced to functional position, i.e., dorsiflexion 90° and valgus 5°. The medial Kirschner wire was further drilled for 3cm and the lateral Kirschner wire further drilled for 2cm. C-arm fluoroscopy was used to confirm whether the ankle reduction and the position of the Kirschner wires were satisfactory. Two 7.5mm cannulated screws (Product model: QWIX, General Care, China) were then percutaneously drilled. Before finishing the surgery, the position of the screws was confirmed with the C-arm fluoroscopy to make sure they were in correct place and did not enter the subtalar joint. Bone graft or joint cavity drainage was not necessary. All incisions were interruptedly sutured with nylon sutures. Plaster cast below the knee joint was used for immobilization.

### 2.5. Postoperative Treatment

Intravenous antibiotics was applied for 24 hours. The injured limb was elevated after surgery. Early exercise without weight-bearing was acceptable. Quadriceps contraction and leg lifting exercises were conducted to prevent muscle atrophy and lower extremity thrombosis. The stitches were taken out 2 weeks postoperatively, and the plaster immobilization was continued to keep the ankle in the functional position. The plaster cast was taken off after 6 weeks; weight-bearing walking could be performed under the protection of walking boots (Aircast, USA) for 4-6 weeks when the radiographs showed bony callus growth. Radiographic examination was conducted 12 weeks postoperatively; if they showed continuous bony callus in the ankle joint space, then the patients could walk with full weight-bearing.

### 2.6. Postoperative Follow-Up Evaluation

American Orthopedic Foot & Ankle Society Ankle Hindfoot Scale (AOFAS) scores were assessed before and 1 year after the surgery. According to the Winson standard [15], the success criteria for fusion is as follows: the ankle is stable and is free of obvious pain when in motion or weight-bearing; radiograph shows that continuous bony callus is formed through the ankle joint without changing the internal fixation and ankle position. Clinical evaluation is divided into 4 grades. “Excellent” means no pain, limping, or occupational limitations; radiograph confirms the fusion. “Good” means minor pain, occasional limping, and some occupational limitations; radiograph confirms the fusion. “Acceptable” means moderate pain, limping, and occupational limitations; radiograph confirms the fusion. “Poor” means significant pain; radiograph confirms unfused condition.

### 2.7. Statistical Analyses

The measured data were shown in mean ± SD and analyzed in SPSS 19.0 statistical software. Student's t-test was used to compare groups and* p* value < 0.05 was considered statistically significant.

This study was approved by the Medical Ethics Committee of Southwest Hospital, and informed consent was obtained from all individual participants included in the study. The research was registered on www.clinicaltrials.gov, numbered NCT03152916.

## 3. Results

Patients were followed for 2.1 ± 0.5 years (1-3 years). There was no significant difference in the degree of ankle deformity between the two groups (*p>*0.05). All of the 29 cases showed bony fusion after surgery. There were no severe complications such as infection or thrombus in both groups. Three cases in the experimental group and two cases in the control group complained of numbness in the incision area, and all cases returned to normal after six months. Detailed results of the follow-up study are shown in Table 1.

**Figure 2:**
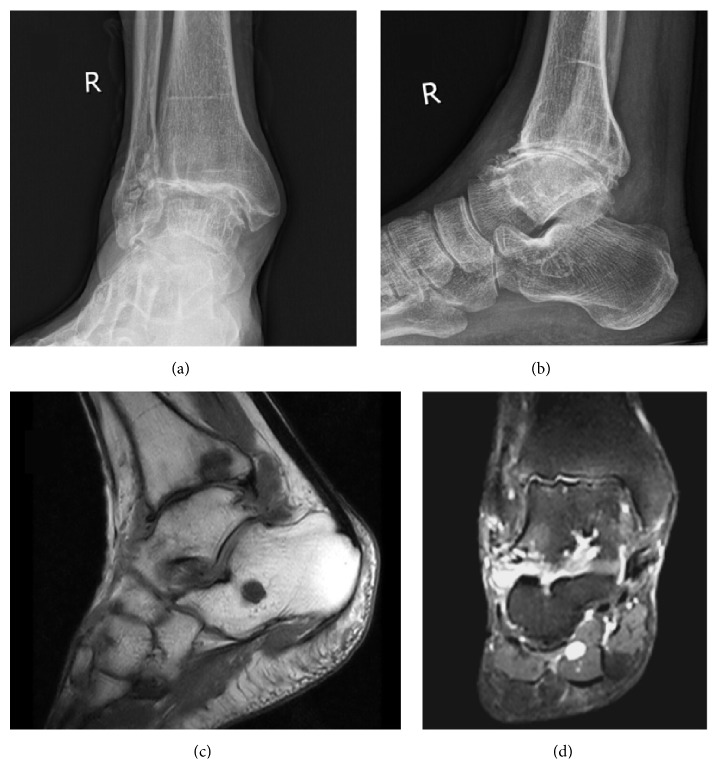
**Preoperative imageological examination suggesting end-stage traumatic arthritis in the right ankle. (a)** Preoperative radiograph of the ankle joint (anteroposterior view).** (b)** Preoperative radiograph of the ankle joint (lateral view).** (c)** Preoperative MRI of the ankle joint (lateral view).** (d) **Preoperative MRI of the ankle joint (anteroposterior view).

**Figure 3:**
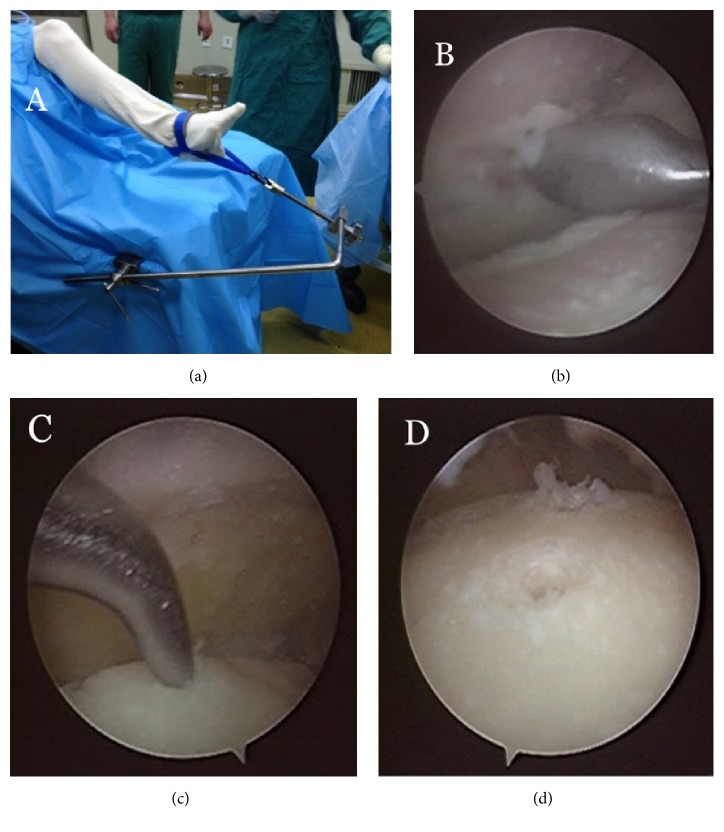
**Arthroscopy. (a)** Position and noninvasive traction.** (b)** Debridement of residual cartilage using curette.** (c)** Microfractures treatment for the fusion surface.** (d)** Articular surface after treatment.

**Figure 4:**
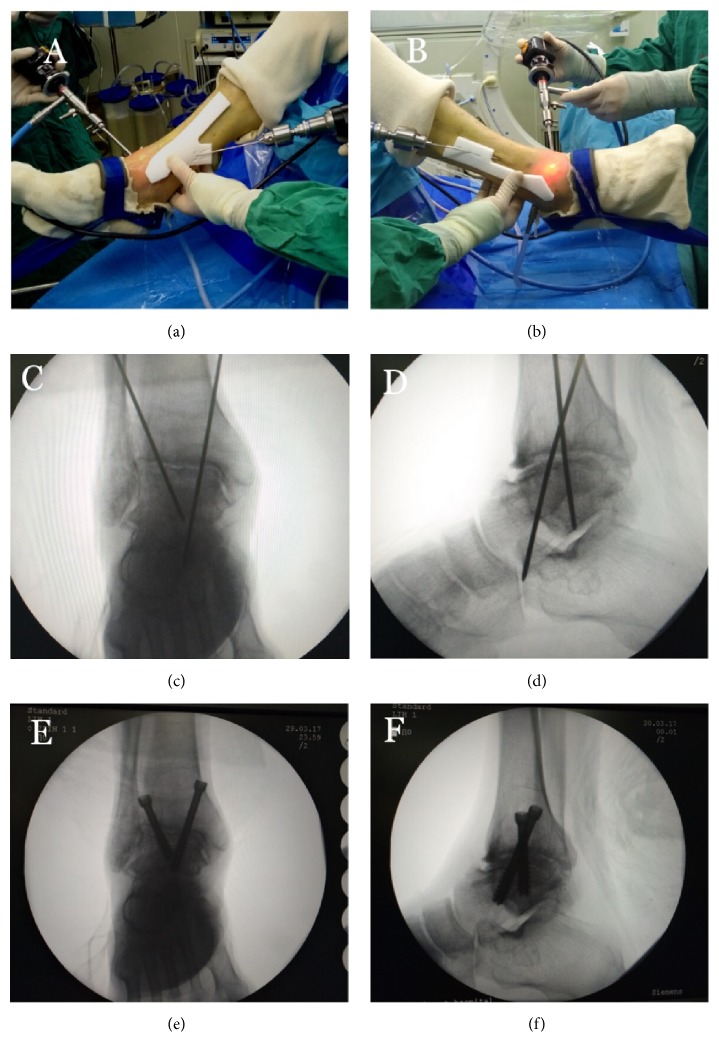
**Ankle arthrodesis assisted by 3D printed personalized guide. (a, b)** Intraoperative operation, drilling of two Kirschner wires with the help of the guides.** (c, d)** C-arm fluoroscopy confirmed satisfactory ankle reduction and Kirschner wire position.** (e, f)** C-arm fluoroscopy showed satisfactory position of the screws.

*Typical Case. *Male, 63 years old, was admitted to the hospital due to “right ankle joint pain with limitation of motion for 2 years”. Conservative treatment in other hospitals failed; right ankle traumatic arthritis was diagnosed preoperatively (Figure 2). The AOFAS scores were 52 points preoperatively and 87 points postoperatively. Key intraoperative techniques included (1) exploration with arthroscope; (2) curettage of articular cartilage residues; (3) refresh of the subchondral bone; (4) microfractures treatment for arthrodesis; (5) drilling of the 2mm Kirschner wires assisted by the medial and lateral personalized guides; (6) drilling the Kirschner wires through the tibiotalar joint space after ankle reduction and confirming the satisfactory position of Kirschner wires with the C-arm fluoroscopy; and (7) percutaneous penetration of the cannulated screws without entering the subtalar joint and finishing the surgery after confirming the position of the screws with C-arm fluoroscopy (Figures 3 and 4).

## 4. Discussion

Ankle arthrodesis was first reported in 1879. Early application of this technique was always associated with higher complications, such as failed bony fusion, poor healing of the incision, blood vessels and nerves injuries, and infections [2, 4]. With the development of the technology, complications of ankle arthrodesis gradually declined, and patients were more satisfied with the outcome. Ankle arthroplasty can be used in some cases with end-stage ankle arthritis, but there is far more ankle arthrodesis than arthroplasty worldwide. Ankle arthroscopy has developed rapidly in recent years, with more ankle diseases treated under arthroscope [16–18]. Ferkel et al. [19] believed that ankle arthrodesis under arthroscope is an in situ fusion that does not correct severe ankle deformities. However, some experienced surgeons also tried to perform arthroscopic ankle arthrodesis for cases that had ankle varus or valgus more than 15° [15].

Arthroscopic ankle arthrodesis is one of the most important basic procedures in foot and ankle surgery [20–28]. The key steps include the following: (1) complete curettage of the articular cartilage is performed; (2) ankle joint is reduced to the functional position; (3) screw fixation is usually applied, and the position and mechanical strength of the screw should be reliable; (4) reasonable rehabilitation training is required after surgery. Previous studies have shown that drilling two crossed cannulated screws in the tibiotalar joint is feasible with higher fusion rate; as the screws are screwed in along the Kirschner wire, the accurate drilling of the Kirschner wire is crucial [4, 29–31]. Often the surgeon drills the Kirschner wire according to his/her previous experience and then uses C-arm fluoroscopy to confirm whether the position of the wire is satisfactory; if not, repeated procedure will be performed. This would lead to an increase in the duration of surgery and intraoperative radiation, which is detrimental to both the surgeon and the patient.

3D printing technology has brought new hope to solve the above problem. The application of 3D printing technology in orthopedic surgery has been rapidly developed and has been successfully applied in many medical fields, such as (1) clearer 3D demonstration of the tissue of the lesion, which is conducive to better communication between the surgeon and the patient as well as the improvement of the surgical plan [13]; (2) preparation of large amount of personalized casts and insoles; (3) preparation of guides that are consistent with the anatomy of the patient to assist in the positioning of the instruments and shorten the duration of the surgery [32]; and (4) printing of personalized metal implants to improve surgical outcomes [12]. In order to prepare the personalized guide, we need to first collect the CT data of the patient to conduct the reconstruction. Software design requires the engineers and technicians to fully understand the purpose of the operation so that the key steps that need the help of the guide can be successfully performed according to the surgeon's intention. The prepared guide should be sterilized before being applied in the surgery. This study suggests that 3D-printed personalized guide designed and prepared based on patient's CT data can simplify the key procedural steps without compromising the surgical outcome. This is a recommendable technique especially for those inexperienced surgeons. The use of personalized guide could reduce the trauma of the operation, shorten the operation time, and reduce the times of using C-arm fluoroscopy during operation, thus reducing the adverse effects of intraoperative radiation.

Though there has been no report of using personalized guides for ankle arthrodesis, our study still has some deficiencies, such as fewer samples and fewer participating units. How to further improve the precise positioning of the guide is also worth further studying. Future multicenter research will be conducted to improve the study.

## Figures and Tables

**Table 1:** Comparative follow-up study of the two groups.

**Grouping**	**Experimental group**	**Control group**	**Statistical analysis (Student's t test)**
Cases	15	14	

Age (years)	56±9	55±11	*p*=0.45

Course of disease (years)	4.2±2.1	3.9±2.2	*p*=0.48

Intraoperative time of positioning the Kirschner wire (minutes)	2.2±0.8	4.5±1.6	*p*=0.001

Intraoperative X-ray scan (times)	2.3±0.1	3.5±0.3	*p*=0.001

Bony fusion time (weeks)	13±1	13±1	*p*=0.82

AOFAS scores at 1 year postoperatively (points)	86±5	85±6	*p*=0.55

Excellent and good rate (%)	93.3	92.9	*p*=0.25

## Data Availability

The data used to support the findings of this study are available from the corresponding author upon request.
